# Supplementary material for: A synthetic growth switch based on controlled expression of RNA polymerase
Source: Mol Syst Biol. 2015 Nov 23;11(11):840. doi: 10.15252/msb.20156382 (PMC4670729; doi:10.15252/msb.20156382)
Supplement: Supplementary file 1 — Appendix [file MSB-11-840-s001.pdf]

Appendix for "A synthetic growth switch based on controlled expression of RNA polymerase"

Jérôme Izard[1,2,+], Cindy D.C. Gomez Balderas[1,2,+], Delphine Ropers[1], Stephan Lacour[1,2], Xiaohu Song[3], Yifan Yang[3], Ariel B. Lindner[3], Johannes Geiselmann[1,2,*], Hidde de Jong[2,*]

1. Université Grenoble Alpes, Laboratoire Interdisciplinaire de Physique (CNRS UMR 5588)
140 rue de la physique BP 87, 38402 Saint Martin d'Hères France.
2. INRIA, Research center Grenoble - Rhône-Alpes, 655 avenue de l'Europe, Montbonnot
38334 Saint Ismier Cedex, France.
3. Center for Research and Interdisciplinarity, INSERM U1001
Medicine Faculty, site Cochin Port-Royal, University Paris Descartes, Sorbonne Paris Cité
24 rue du Faubourg Saint Jacques, 75014 Paris, France.

[+] Both authors contributed equally to this work.
[*] Corresponding authors with equal contributions:
Johannes Geiselmann (Hans.Geiselmann@ujf-grenoble.fr),
Hidde de Jong (Hidde.de-Jong@inria.fr)

Contents

Appendix Figure S1. Construction of R strain: inducible expression of *rpoBC* genes. **3**

Appendix Figure S2. Construction of R strain: overexpression of *lacI* from additional copies on chromosome. **4**

Appendix Figure S3. Construction of W-*rpoC*-mCherry and R-*rpoC*-mCherry strains: translational fusion of *rpoC* and fluorescent reporter. **5**

Appendix Figure S4. Construction of W-gly and R-gly strains: plasmid for glycerol production. **6**

Appendix Text S5. Analysis of fluorescent reporter gene data. **7**

Appendix Text S6. Analysis and processing of microfluidics imaging data. **10**

Appendix Figure S7. Computation of growth rate of individual R cells in the microfluidics device. **12**

Appendix Figure S8. Effect of external control of *rpoBC* gene expression on cell size, quantified by means of fluorescence and phase-contrast microscopy. **13**

Appendix Figure S9. Glycerol consumption and production in W strain and growth-arrested R strain. **14**

Appendix Table S1. Primers used for strain construction. **15**

Appendix Figure S1 – Construction of R strain: inducible expression of *rpoBC* genes

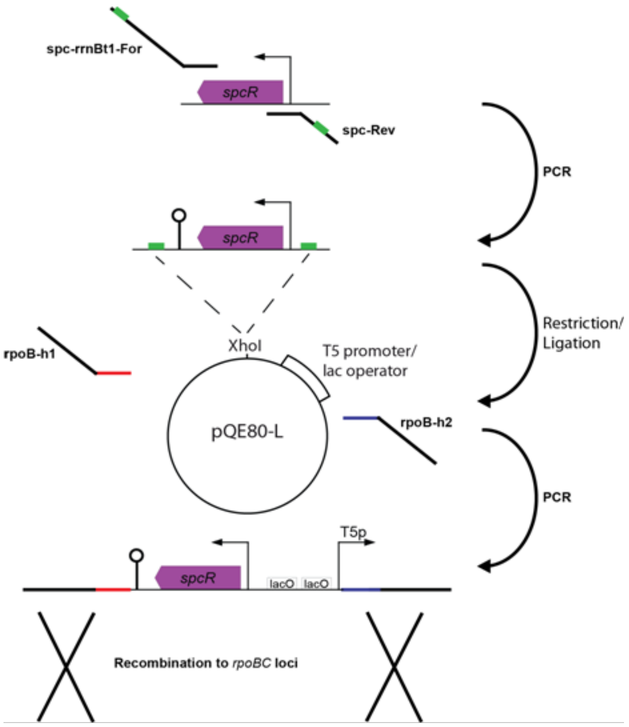

A spectinomycin-resistance (*spcR*) cassette was PCR-amplified from the pGBM2 plasmid using primers spc-rrnBt1 and spc-Rev (Appendix Table S1). They both carry a *XhoI* restriction site, and spc-Rev also contains a strong, bidirectional terminator. We cloned this cassette upstream of the inducible T5 promoter of the pQE80-L plasmid (Qiagen). The promoter contains two *lac* operator sequences that ensure efficient repression by the *lac* repressor (Lutz and Bujard, 1997). Using primers rpoB-h1 and rpoB-h2 (Appendix Table S1), we PCR-amplified the region spanning the *rrnBT1* terminator, the *spcR* cassette and the T5 pQE80-L promoter. This PCR product was recombined upstream of the *rpoBC* coding sequence (in place of the 320 bp DNA region between *rplL* and *rpoBC*). Sequences can be obtained upon request.

Appendix Figure S2 – Construction of R strain: overexpression of *lacI* from additional copies on chromosome

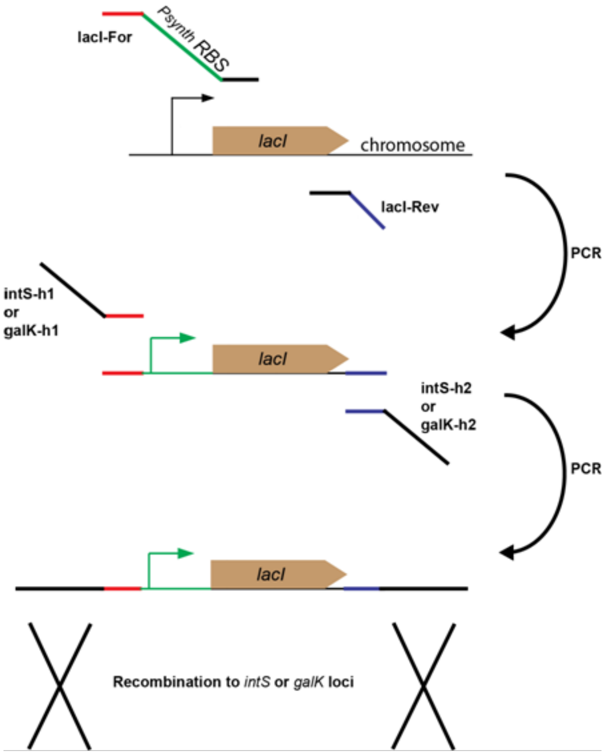

In order to ensure a high expression of the *lac* repressor, we introduced a strong synthetic promoter (Braatsch *et al*, 2008) into the forward primer lacI-For. The *lacI* gene was PCR-amplified using primers lacI-For and lacI-Rev. We recombined the PCR product in replacement of the *galK* and *intS* genes by using a *ccdB*-toxin counterselection system (Ranquet *et al.*, manuscript in prepration) without leaving an antibiotic resistance cassette on the chromosome. The sequences of the primers are listed in Appendix Table S1.

Appendix Figure S3 – Construction of W-*rpoC*-mCherry and R-*rpoC*-mCherry strains: translational fusion of *rpoC* and fluorescent reporter

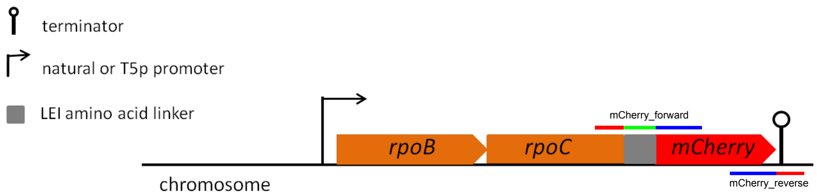

We fused the mCherry, red fluorescent protein to the C-terminus of the β' subunit of RNA polymerase, encoded by the *rpoC* gene. Three additional amino acids, LEI, coded by the sequence CTAGAAATA, serve as a linker between the two proteins. The native terminator of the *rpoBC* operon is therefore situated just downstream of mCherry. Primers mCherry_forward and mCherry_reverse were used to amplify the mCherry gene and sequences overlapping the end of *rpoC*. The DNA fragment amplified in this way was inserted into the chromosome using a *ccdB*-counterselection cassette as indicated in Appendix Figure S2. Primers rpoC_left and rpoC_right were used to verify the sequence on the chromosome. The complete sequences can be obtained upon request.

Appendix Figure S4 – Construction of W-gly and R-gly strains: plasmid for glycerol production

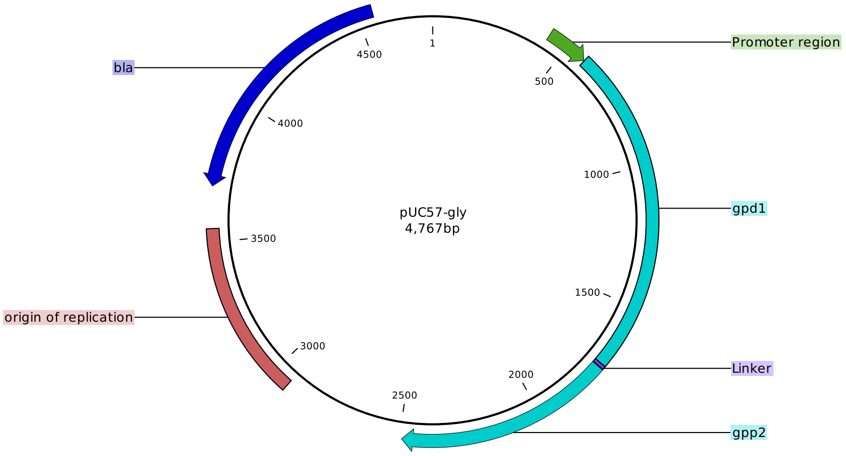

The pUC57-gly plasmid carries a fusion of the *Saccharomyces cerevisiae* genes *GPP2* and *GPD1*, whose product catalyzes the conversion of dihydroxyacetone phosphate to glycerol (Liang *et al*, 2011). The sequence of the gene has been codon-optimized for *E. coli* by means of the OptimumGene® tool (GenScript). Its transcription is under the control of the *rpsM* promoter (Post *et al*, 1980), a strong and constitutive promoter with an activity that was found to be relatively constant over kinetics experiments of the type described in Figure 2*A* in the main text, as verified by means of a fluorescent reporter gene (Zaslaver *et al*, 2006). The W and R strains were both transformed with the pUC57-gly plasmid, giving rise to the glycerol production strains labeled W-gly and R-gly, respectively. The genetic constructions were confirmed using the primer set gpd1-gpp2_F/ gpd1-gpp2_R, listed in Appendix Table S1. The plasmid sequence can be obtained upon request.

Appendix Text S5 – Analysis of fluorescent reporter gene data

As described in the *Materials and methods* section of the main text, the amount of the β' subunit of RNA polymerase was quantified by means of a fluorescent tag using the W-*rpoC*-mCherry and R-*rpoC*-mCherry strains. In order to interpret these data, we used the following model adapted from de Jong *et al* (2010) and references therein.

$$\frac{d}{dt} r_m(t) = k_m \left(r_t(t) - r_m(t) \right) - \left(\mu(t) + \gamma \right) r_m(t), \tag{S1}$$

$$\frac{d}{dt} r_t(t) = f(t) - \left(\mu(t) + \gamma \right) r_t(t), \tag{S2}$$

where r_m, r_t [mM] represent the concentrations of mature and total (folded) mCherry in the cell population, respectively, which vary with time t. μ [min^{-1}] is the growth rate, γ [min^{-1}] the degradation constant, k_m [min^{-1}] the maturation constant, and $f(t)$ [mM min^{-1}] the synthesis rate of mCherry. The model describes the balance between the synthesis and decay of total and mature mCherry, where the decay includes both physical degradation and growth dilution. The rate of maturation is proportional to the concentration of immature (unfolded) mCherry, equal to the difference between the concentrations of total and mature mCherry. Notice that only mature mCherry is observed, whereas we are interested in reconstructing the concentration of total mCherry (and thus of β').

We further define, as usual,

$$\mu(t) = \frac{1}{V(t)} \frac{d}{dt} V(t), \tag{S3}$$

where $V(t)$ [L] is the total volume of the growing cell population, and define

$$r_m(t) = R_m(t)/V(t), \qquad r_t(t) = R_t(t)/V(t), \tag{S4}$$

where R_m, R_t [mol] represent the molar quantities of mature and total mCherry in the population, respectively. The volume is estimated from measurements of the absorbance at 600 nm and the molar quantity of mature mCherry from the fluorescence measurements. That is, we have

$$\hat{V} = \alpha V + \epsilon, \qquad \hat{R}_m = \beta R_m + \nu, \tag{S5}$$

where α, β are unknown proportionality constants and ϵ, ν measurement noise. In what follows, we arbitrarily set α and β to 1, which means that we estimate the volume and the amount of mCherry, and thus the mCherry concentration, up to a proportionality constant, as is usual for reporter gene measurements (de Jong *et al*, 2010).

In order to estimate k_m and γ, we performed a calibration experiment where protein synthesis in exponential growth is stopped by adding the antibiotic choramphenicol (0.3 g/L). The experiment is identical to the growth experiments reported in Figure 2*A* of the main text and carried out in several growth media, namely M9 with 0.2% glucose, M9 with 0.2% glucose and 0.2% casamino acids, and LB with 0.2% glucose. Choramphenicol was added in mid-exponential phase to the growing bacterial culture, and the fluorescence signal recorded. The accumulation of fluorescence reflects the maturation of mCherry only, since the addition of choramphenicol has blocked the synthesis of new mCherry molecules. The fluorescence signal reaches a plateau once all mCherry has been converted to its mature form. An example of a typical fluorescence time-course in M9 medium with glucose is shown in Appendix Figure S5.1*A*.

We analyzed the data by a variant of the model of Eqs. S1 and S2, where $f(t)$ is set to 0 to represent the absence of further protein synthesis after chloramphenicol addition. Using the definitions of r_m and r_t, the model can be reformulated as follows:

$$\frac{d}{dt}R_m(t) = k_m\,(R_t(t) - R_m(t)) - \gamma\,R_m(t), \tag{S6}$$

$$\frac{d}{dt}R_t(t) = -\gamma\,R_t(t), \tag{S7}$$

and this model was fit against the data over a time-interval in which all mCherry present at the time of choramphenicol addition was converted to its mature form (the red data points in Appendix Figure S5.1*A*). The fit of the model in Eqs. S6 and S7 is also shown in the plot. This resulted in a k_m value equal to 0.008 min^{-1} in M9 medium with glucose, 0.006 min^{-1} in M9 medium with glucose and casamino acids, and 0.009 min^{-1} in LB medium with glucose, corresponding to maturation times of around 85 min, 115 min, and 60 min, respectively, where the maturation time is defined as $\ln 2/k_m$. The reported values are the mean of 10-15 replicates, using the strains W-*rpoC*-mCherry and R-*rpoC*-mCherry with 1000 μM IPTG. Notice that different media lead to different estimates of the maturation constants, in accordance with the observations in Hebisch *et al* (2013). However, contrary to these authors, an overall good fit was obtained while assuming exponential maturation kinetics. In all growth media a degradation constant of around 0.0045 min^{-1} was obtained, corresponding to a half-life of around 25 h. This is consistent with the high stability of mCherry reported in the literature (Hebisch *et al*, 2013). Appendix Figure S5.1*B* shows another example fit, for an experiment carried out in M9 with glucose and casamino acids.

In order to compute the mCherry concentration, we used the model of Eq. S1 together with the values for k_m and γ obtained in the calibration experiment. Like in the calibration experiment, the model was slightly formulated using the definitions of r_m and r_t, giving rise to

$$R_t(t) = \frac{1}{k_m}\,\frac{d}{dt}R_m(t) + \frac{k_m + \gamma}{k_m}\,R_m(t) \tag{S8}$$

This allows an estimate of the time-varying total amount of mCherry, $R_t(t)$, to be inferred from the observed quantity of mature mCherry, using a smoothing spline fit (function `csaps` in Matlab) of the fluorescence signal. The estimate of the concentration $r_t(t)$ is then obtained by dividing the estimate of $R_t(t)$, computed from Eq. S8, by the measured absorbance. Appendix Figure S5.1*C*-*D* show an example (fit) of the fluorescence and absorbance signals in the interval in which the growth rate is computed as well as the mCherry concentration computed from this information, following the procedure outlined above. For each replicate experiment, we computed the mean of the mCherry concentration over this interval, while the reported mCherry concentrations in Figure 3 in the main text is the mean obtained from 5 replicate experiments.

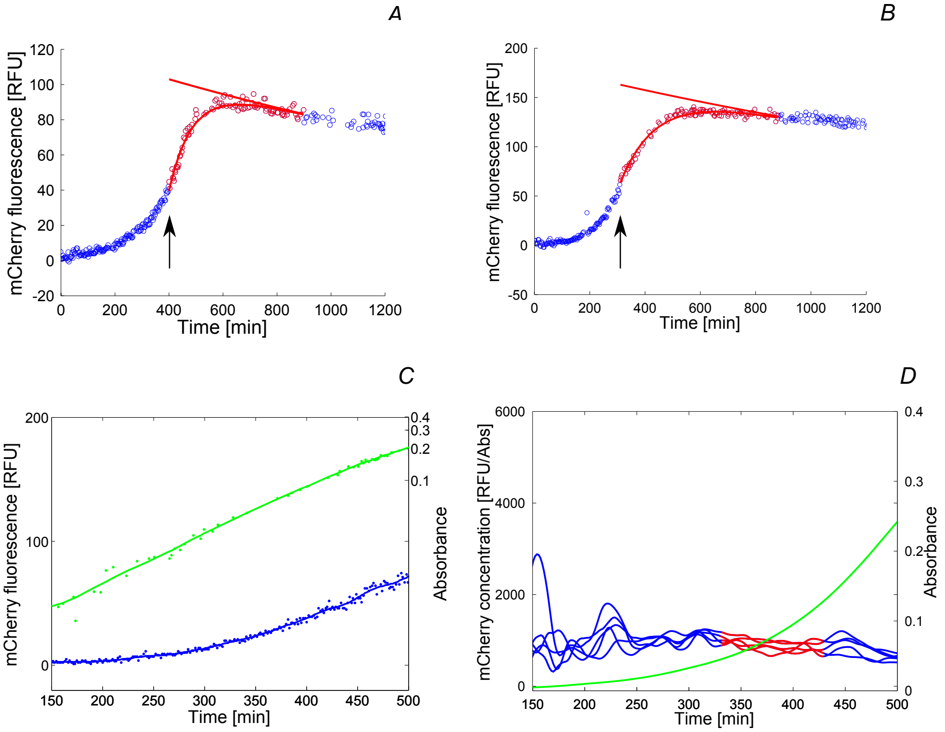

Appendix Figure S5.1: Illustration of data analysis procedures for computation of mCherry concentration. *A:* Calibration of the reporter gene expression model. mCherry fluorescence (○, blue) is measured in a reporter gene experiment using the W-*rpoC*-mCherry strain growing in M9 medium supplemented with 0.2% glucose. Chloramphenicol is added to the growing culture, causing the arrest of protein synthesis (arrow). The maturation constant k_m and degradation constant γ are determined by fitting the model of Eqs. S6 and S7 to the red data points. The two curves show the fit of the quantity of mature mCherry against the fluorescence data as well as the predicted total amount of mCherry. The plot shows one of the 10-15 replicates used to estimate k_m and γ. *B:* Idem, for W-*rpoC*-mCherry strain growing in M9 medium supplemented with 0.2% glucose and 0.2% casamino acids. *C:* Fluorescence (○, blue) and absorbance (○, green) data acquired in reporter gene experiment using W-*rpoC*-mCherry strain growing in M9 medium supplemented with 0.2% glucose, under the conditions shown in Figure 2 of the main text. The plot also shows the fit of a smoothing spline to the data (solid lines). *D:* Computation of mCherry concentration in the interval in which growth rates reported in Figure 2 in the main text have been computed (red curve segments). Note that in this interval the mCherry concentrations are approximately constant. We show the computed mCherry concentration for five replicates.

Appendix Text S6 – Analysis and processing of microfluidics imaging data

We used a variant of the mother machine (Wang *et al*, 2010) to extract analyzable information about single cells (Gasset-Rosa *et al*, 2014). The images were obtained every 2 to 5 min using time-lapse microscopy. Each image (position) contains about 18 to 30 channels depending on the microscope magnification (Zeiss 63x or Nikon 100x). Typical sampling resolutions along the x and y directions were 0.9-1.4 μm.

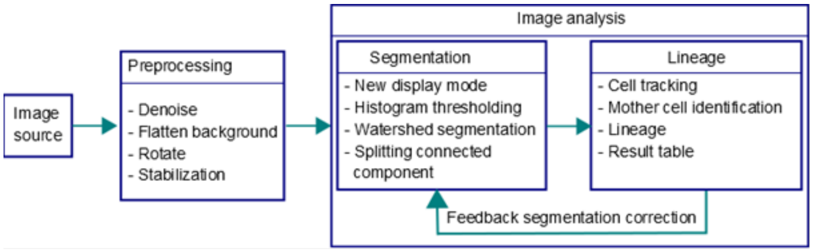

Appendix Figure S6.1: Flow diagram of image processing pipeline.

A flow diagram of image processing is shown in Appendix Figure S6.1. Our image preprocessing procedures were selected for maximum simplicity and efficiency. The preprocessed images were stored and used for cell segmentation and lineage. A median filter with a kernel width of 3 was applied to each slice of the original image to suppress the effects of shot noise. As a side-effect of the mother machine structure, the light was uneven over the whole image as shown in Appendix Figure S6.2. The uneven background was estimated by a Close-Open morphology filter, using an anisotropic kernel, and subtracted from the image.

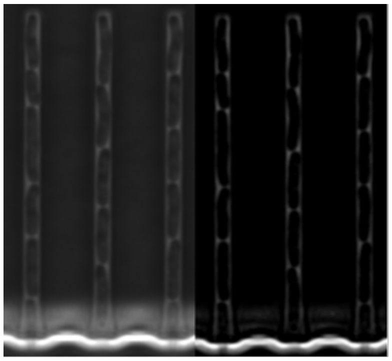

Appendix Figure S6.2: Uneven light and preprocessed image.

The images were then rotated manually by alignment on predefined grid lines whereby the rotation angle was estimated from one slice (often the first image). The rotated images were then subjected to framing with an estimation of the region of interest by computing the biggest sum intensity of the horizontal edge using least squares. The resulting images were then stabilized by the Lucas-Kanade algorithm (Lucas and Kanade, 1981).

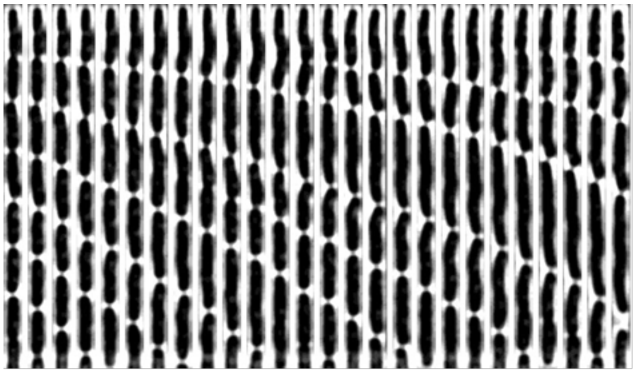

Appendix Figure S6.3: Innovative display of cell tracking.

The net result from the image preprocessing procedures noted above is an initial extraction of information for each channel. For the study of cell lineage, each channel slice was cut and pasted on a new image, from left to right according to the acquisition time (Appendix Figure S6.3). This treatment yields information about cell lineage. For cell segmentation, an intensity-based segmentation was applied first. The selected threshold level was user-defined and may contain under-segmented (connected) components arising from image artifacts that must be manually split as appropriate. Watershed segmentation (Meyer, 1991) was then applied for separating the connected components after the binary image was transformed on a distance map. Each watershed line was then attributed to an intensity measured on the vertical gradient image. The intensities were then inserted into a clustering model (Otsu, 1979) that generates an automatic level by maximizing the variance of two classes. These merging and splitting decisions are based on a simple 1D mathematical model of the cell based on the edge feature. This model can be constructed automatically and adaptively from the initial segmentation results. The error rate of the segmentation was between 0.1-1%.

Within the obtained regions of interest, the lineage was studied by the length of corresponding cells. A cell is considered a mother cell if its length is larger than the length of the cell in the next image. Therefore, in the absence of segmentation errors, the lineage could be performed without any difficulties. Interestingly, as noted above, some segmentation errors that are not immediately obvious show up in this display mode (Appendix Figure S6.4). Feedback from the lineage results thus greatly simplifies error correction. The user can choose to automatically correct the segmentation error in the lineage process or correct the error manually (as the lineage error is below 1%). The final result is stored into a result table for further analysis by Matlab or R.

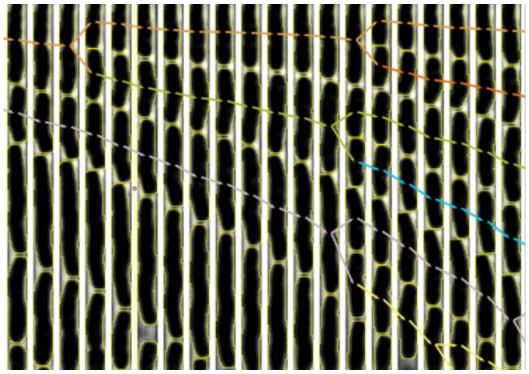

Appendix Figure S6.4: Feedback from the lineage process.

Appendix Figure S7 – Computation of growth rate of individual R cells in the microfluidics device

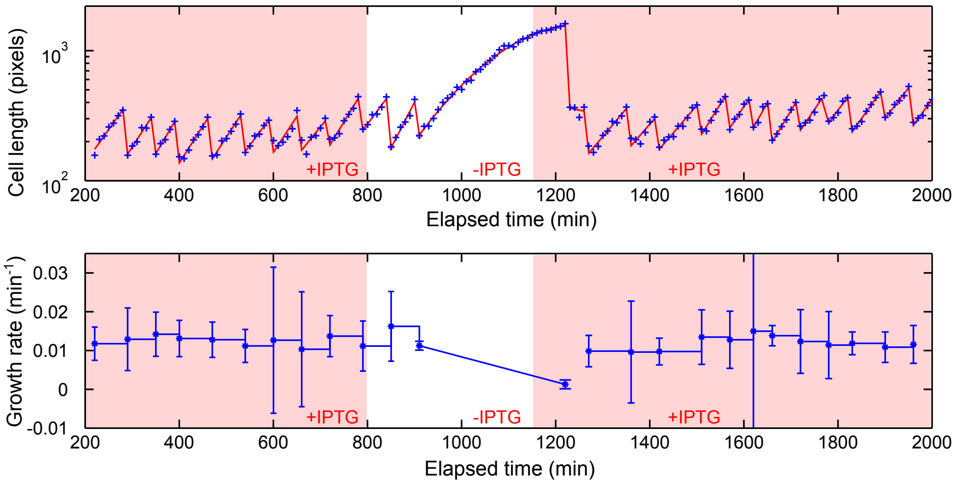

Bacteria trapped in the channels of the microfluidics device were grown in M9 minimal medium with 0.2% glucose, with or without 1000 μM IPTG. The growth rate of R cells and their descendants was quantified by measuring the cell length of the newly-formed bacteria in successive frames of time-lapse microscopy. Measurements of the cell length of a mother cell and its progeny are given in blue in the upper panel. The vertical lines denote the time of addition and removal of IPTG. The growth rate of individual exponentially-growing cells was obtained by fitting an exponential to the cell-length data. In the case of filamentous cells, a second-order polynomial was used to fit the natural logarithm of the cell length: $\log L = a \cdot t^2 + b \cdot t + c$, with cell length L, time t and parameters a, b, c. The time derivative of this polynomial gives the (time-varying) growth rate of the filamentous cell: $\mu(t) = 2 \cdot a \cdot t + b$. The fit of the different growth curves for an individual cell and its descendants is shown in red in the upper panel. The corresponding growth rates are shown in the lower panel, along with their 95% confidence intervals. Figure 4 in the main text is obtained by combining data from 100 analyses of the type shown in this figure.

Appendix Figure S8 – Effect of external control of *rpoBC* gene expression on cell size, quantified by means of fluorescence and phase-contrast microscopy

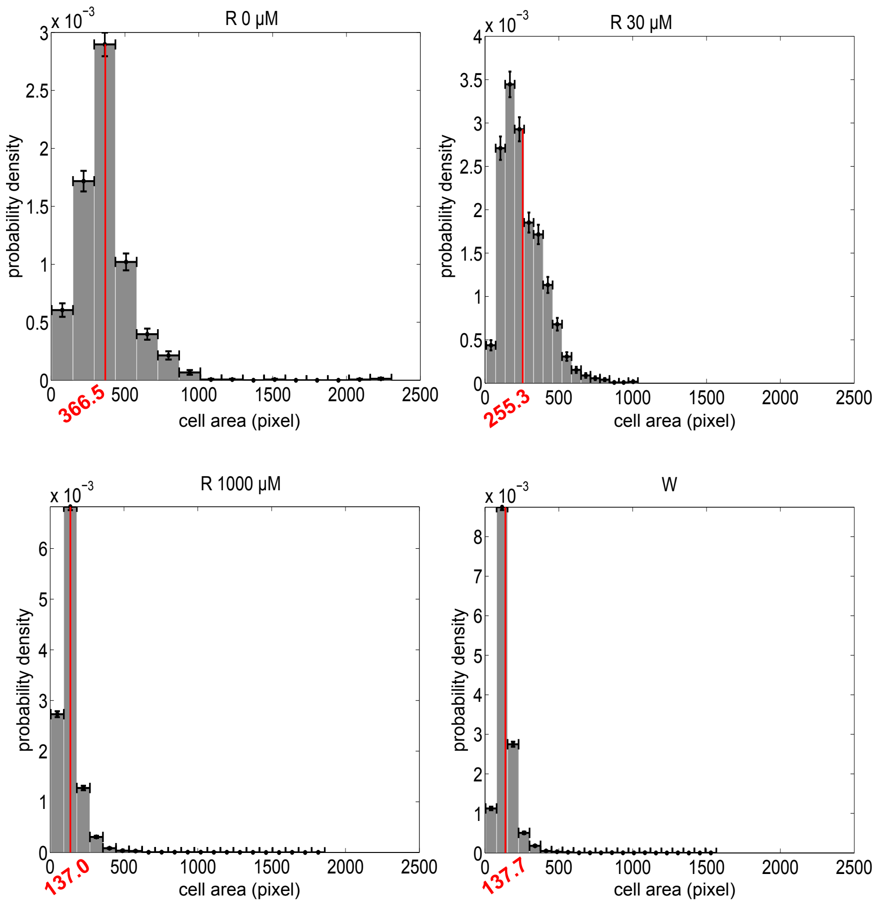

W and R cells were sampled after 5 hours of growth in M9 medium with 0.2% glucose and different concentrations of IPTG in a shake flask, stained with Hoechst dye, fixed and visualized by fluorescence and phase-contrast microscopy. Between 1200 cells (R strain with 0 μM IPTG) and 8000 cells (R strain with 1000 μM and W strain) were imaged and their cell area was calculated using the Scan® software of Olympus. Although the fluorescent dye specifically binds to DNA, the background fluorescence was sufficiently high to prevent segmentation of cells with multiple nuclei. The four histograms show the distribution of cell areas for the four cultures considered (R strain with 0, 30, or 1000 μM IPTG and W strain with no IPTG). The red line indicates the mean cell area. Since no changes in cell width are observed, the cell volume is considered proportional to the cell area. The optimal number of bins for each histogram was determined by means of Doane's rule (Doane, 1976).

Appendix Figure S9 – Glycerol consumption and production in R strain

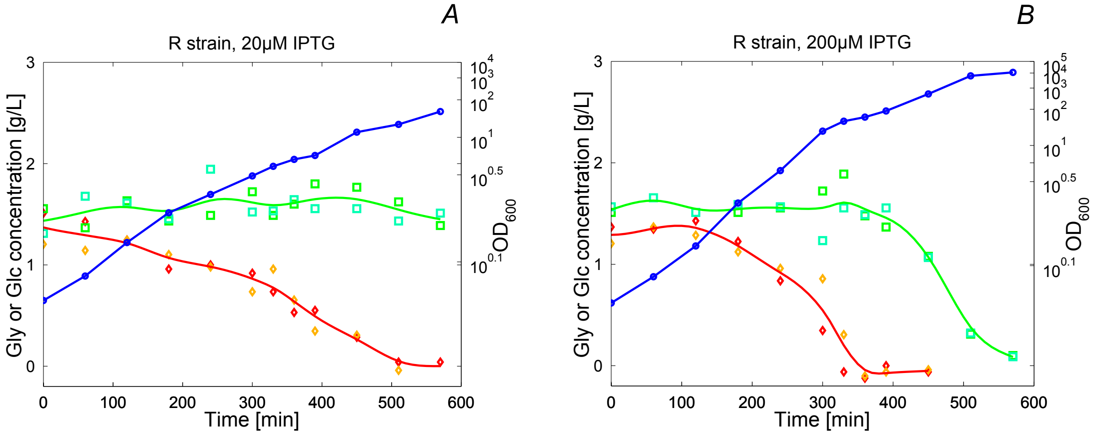

The glycerol consumption and production patterns were quantified for the R strain growing in shake flasks in M9 medium supplemented with 0.2% glucose and (*A*) a low concentration of IPTG (20 μM) and (*B*) a high concentration of IPTG (200 μM). The quantification of glycerol (green and turquoise data points) and glucose (red and orange data points) was achieved as described in the *Materials and methods* in the main text. The data points come from two replicate experiments. The red and green curves are spline fits indicating the trend of the glucose and glycerol concentrations. The blue data points are OD_{600} measurements (only one replicate shown). The data confirm that, like for the W strain, glycerol is not produced during growth on glucose and not consumed before exhaustion of glucose, as expected from the effect of carbon catabolite repression. These results show that in our conditions the natural glycerol consumption and production pathway of *E. coli* does not interfere with the engineered pathway carried on the pUC57-gly plasmid (Appendix Figure S4).

Appendix Table S1 – Primers used for strain construction

Primer name	Primer sequence
spc-rrnBt1-For	ACGTCTCGAGATAAAACGAAAGGCTCAGTCGAAAGACTGGGCCTTTCGTTTTATA ATTGTTAGACATTATTTGC
spc-Rev	CTGATAGGGACTCGAGCCAG
rpoB-h1	AGCCTTTTTGCGCTGTAAGGCGCCAGTAGCGTTTCACACTGTTTGACTACTGCTG TGCCTGGCCCTTTCGTCTTCACCTC
rpoB-h2	TTGTGGACGTTTACCAAAATCCTTACGAATACGTTTTTTCTCGGTATAGGAGTAAA CCATAGTTAATTTCTCCTCTTTAA
lacI-For	TAAGCTAGCGTGACGATGCGTTGACATATCACTGTGATTCACATATAATATGCGAA ATCAGAAGAGTATTGCTAATGAAACCAGTAACGTTATA
lacI-Rev	TAACTAGCTGATCACCTTAGTCACTGCCCGCTTTCCAGTC
intS_h1	CCGTAGATTTACAGTTCGTCATGGTTCGCTTCAGATCGTTGACAGCCGCATAAGC TAGCGTGACGATGCG
intS_h2	ATAGTTGTTAAGGTCGCTCACTCCACCTTCTCATCAAGCCAGTCCGCCCATAACT AGCTGATCACCTTAG
galK_h1	GTTTGCGCGCAGTCAGCGATATCCATTTTCGCGAATCCGGAGTGTAAGAATAAGC TAGCGTGACGATGCG
galK_h2	TTCATATTGTTCAGCGACAGCTTGCTGTACGGCAGGCACCAGCTCTTCCGTAACT AGCTGATCACCTTAG
mCherry_forward	CCAGCCTGGCAGAACTGCTGAACGCAGGTCTGGGCGGTTCTGATAACGAGCTAG AAATAATGACTAGCAAAAGATCCAAG
mCherry_reverse	CCCCCCATAAAAAAACCCGCCGAAGCGGGTTTTTACGTTATTTGCGGATTATTAT TTGTACAGCTCATCCATG
gpd1-gpp2_F	TCGCATATTTTTCTTGCAAA
gpd1-gpp2_R	TCACCATTTCAGCAGGTC
F-delta LacY	TCGGTCATTGGCATG
R-delta LacY	TGGTCTGGTGTCAAAAATAA
rpoC_left	AAGAGAACGTTATCGTGGGT
rpoC_right	GACAAATGCTCTTTCCCTAA

The table lists all the primers used to construct the W and R strains. Highlighted nucleotides correspond to sequences homologous to the amplified fragments (blue, green, and red) mentioned in Appendix Figures S1-S3. The primers gpd1-gpp2_F and gpd1-gpp2_R have been used to verify the W-gly and R-gly strains, the primers F-delta LacY and R-delta LacY to verify the W-$\Delta lacY$ and R-$\Delta lacY$ strains, and the primers rpoC_left and rpoC_right to verify the W-*rpoC*-mCherry and R-*rpoC*-mCherry strains.

References

Braatsch S, Helmark S, Kranz H, Koebmann B, Jensen PR (2008) *Escherichia coli* strains with promoter libraries constructed by Red/ET recombination pave the way for transcriptional fine-tuning. *Biotechniques* **45**: 335–7

de Jong H, Ranquet C, Ropers D, Pinel C, Geiselmann J (2010) Experimental and computational validation of models of fluorescent and luminescent reporter genes in bacteria. *BMC Syst Biol* **4**: 55

Doane DP (1976) Aesthetic frequency classifications. *Am Statist* **30**: 181–183

Gasset-Rosa F, Coquel AS, Moreno-Del Álamo M, Chen P, Song X, Serrano AM, Fernández-Tresguerres ME, Moreno-Díaz de la Espina S, Lindner AB, Giraldo R. (2014) Direct assessment in bacteria of prionoid propagation and phenotype selection by Hsp70 chaperone. *Mol Microbiol* **91**: 1070–87

Hebisch E, Knebel J, Landsberg J, Frey E, Leisner M (2013) High variation of fluorescence protein maturation times in closely related *Escherichia coli* strains. *PLoS One* **8**: e75991

Liang Q, Zhang H, Li S, Qi Q (2011) Construction of stress-induced metabolic pathway from glucose to 1,3-propanediol in *Escherichia coli*. *Appl Microbiol Biotechnol* **89**: 57–62

Lucas B, Kanade T (1981) An iterative image registration technique with application to stereo vision. *Proc 7th Int Joint Conf Artif Intell (IJCAI-81)*, Vancouver, BC. 674-679

Lutz R, Bujard H (1997) Independent and tight regulation of transcriptional units in *Escherichia coli* via the LacR/O, the TetR/O and AraC/I1-I2 regulatory elements. *Nucleic Acids Res* **25**: 1203–10

Meyer F (1991) Un algorithme optimal pour la ligne de partage des eaux. *Actes 8ème congrès de reconnaissance des formes et intelligence artificielle*, Lyon, France. 847-857

Otsu N (1979) A threshold selection method from gray-level histograms. *IEEE Trans Syst Man Cybernet* **9**: 62-66

Post LE, Arfsten AE, Davis GR, Nomura M (1980) DNA sequence of the promoter region for the α ribosomal protein operon in *Escherichia coli*. *J Biol Chem* **255**: 4653–9

Wang P, Robert L, Pelletier J, Dang WL, Taddei F, Wright A, Jun S (2010) Robust growth of *Escherichia coli*. *Curr Biol* **20**: 1099–103

Zaslaver A, Bren A, Ronen M, Itzkovitz S, Kikoin I, Shavit S, Liebermeister W, Surette MG, Alon U (2006) A comprehensive library of fluorescent transcriptional reporters for *Escherichia coli*. *Nat Methods*, 3: 623–8
