# Supplementary material for: A synthetic growth switch based on controlled expression of RNA polymerase
Source: Mol Syst Biol. 2015 Nov 23;11(11):840. doi: 10.15252/msb.20156382 (PMC4670729; doi:10.15252/msb.20156382)
Supplement: Supplementary file 2 — Expanded View Figures PDF [file MSB-11-840-s002.pdf]

Expanded View Figures

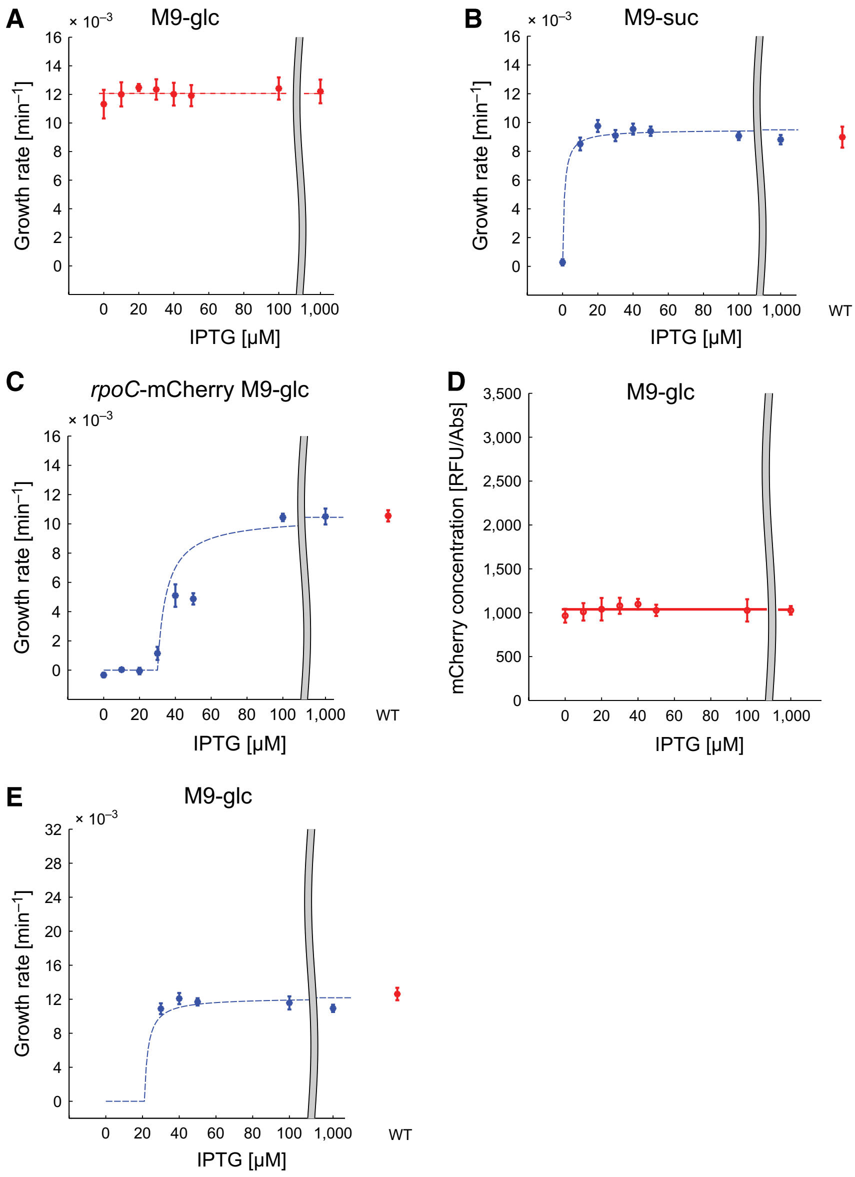

Figure EV1. Growth rate and mCherry concentration in additional strains and conditions.

Additional controls of the IPTG dependence of the growth rate in the W and R strains, with and without the mCherry tag on *rpoC*. All experiments have been carried out in the conditions summarized in Fig 2 including the number of replicates and data treatment.

- A The growth rate of the W strain in M9 medium with 0.2% glucose does not vary with the concentration of IPTG.
- B Variation of the growth rate with IPTG concentration in the R strain grown in M9 minimal medium with 0.2% succinate, using the W strain as a control. The maximum growth rate attained is 0.009 $\pm$ 0.0007 min^{-1}, corresponding to a doubling time of 77 min. 10 μM IPTG is sufficient for growth, consistent with the lower growth rate supported by this medium in comparison with the reference medium, M9 minimal medium supplemented with glucose.
- C Variation of the growth rate with the IPTG concentration in the R-*rpoC*-mCherry strain grown in M9 medium with 0.2% glucose, with the W-*rpoC*-mCherry strain as a control (in red). The switching phenotype is the same as in the R strain.
- D The concentration of mCherry as a function of IPTG in the W-*rpoC*-mCherry strain, grown in M9 medium with 0.2% glucose. In a strain in which *rpoBC* expression is controlled by the natural promoter, the mCherry concentration does not vary with the IPTG concentration, as expected.
- E The same experiment as in Fig 2B, but with a 10 times higher dilution rate at inoculation, giving an initial OD_{600} of 0.001. The curve shown in the plot is the same as in Fig 2B. The essential observation is that, like for an initial OD_{600} of 0.01, the switching threshold lies between 20 and 30 μM. Since for a 10 times higher dilution rate, the absorbance of growth-arrested cultures is so low that it cannot be distinguished from the background absorbance, the growth rates for IPTG concentrations between 0 and 20 μM have not been computed.

Source data are available online for this figure.

© 2015 The Authors

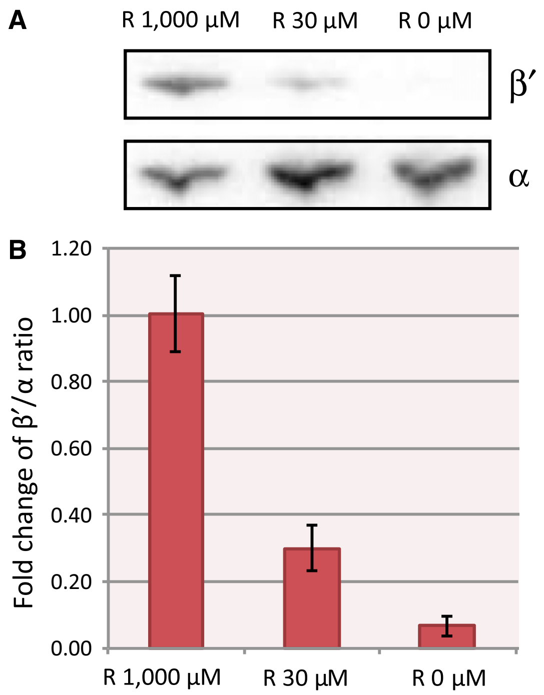

Figure EV2. Western blots to quantify RNA polymerase subunits.

A Images of Western blots of RNA polymerase α and β' subunits in the R strain, acquired as described in the Materials and Methods section.

B Relative concentrations β'/α, normalized to the value of this ratio in the R strain with 1,000 µM IPTG (Materials and Methods). The reported values are the mean of four independent experiments and the error bars show the standard deviation. Weak or no induction of the expression of the *rpoBC* operon leads to substantially lowered relative concentration β'/α. This indicates that, when decreasing the expression of the *rpoBC* operon in the R strain, the β' subunit of RNA polymerase becomes limiting.

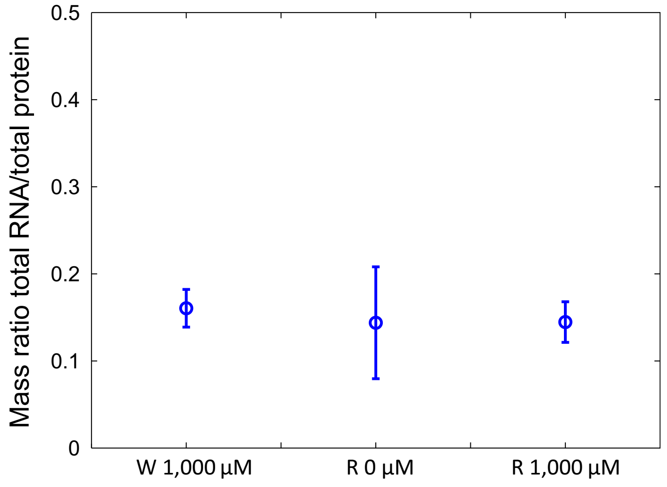

Figure EV3. Quantification of total RNA and protein.

Mass ratio of total RNA and protein for different concentrations of IPTG, quantified by spectrophotometry and by Bradford assays, respectively, as described in the Materials and Methods section. The reported values are the mean of 9–12 replicates and the error bars represent ± two standard errors of the mean. The RNA/protein mass ratio is the same, within the limits of experimental error, in the W strain and in the R strain with 0 and 1,000 µM IPTG.

Source data are available online for this figure.

© 2015 The Authors

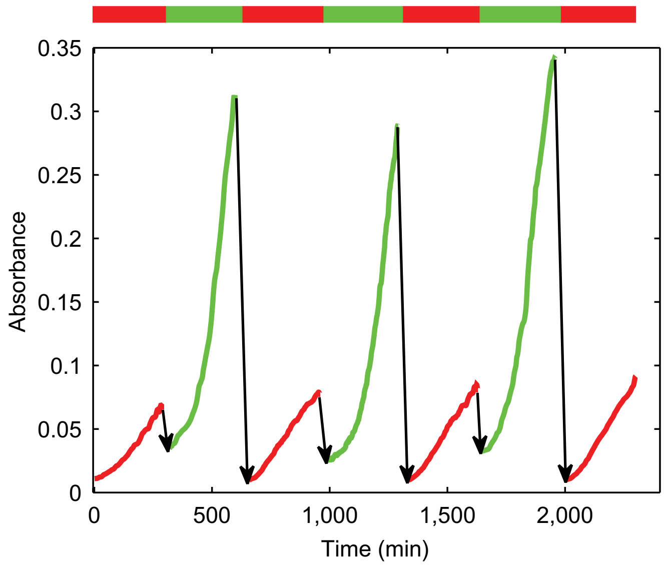

Figure EV4. Repeated reversibility of growth arrest.
The R strain was grown in a microplate as described in the Materials and Methods section, in M9 minimal medium with 0.2% glucose, and alternating between a high and a low-IPTG concentration (1,000 μM vs. 10 μM). The bars on top of the panel indicate the concentration of IPTG (red: low, green: high). The plot shows the mean absorbance at 600 nm over four repeated cycles (including the preculture phase). The mean is computed from four replicates. Notice that growth recovers very rapidly after transfer to a high-IPTG medium (there is an approximate delay of 30 min between samples due to washing and transfer of the samples, indicated by the arrows). Like in Fig 2A, growth continues (though at an increasingly lower rate) after transfer to a low-IPTG medium, until the RNA polymerase present in the cells from the previous high-IPTG growth phase have been diluted out. The kinetics and the extent of the decrease in growth rate after removal of IPTG are identical to what is observed in the microfluidics experiment of Fig 4.

 © 2015 The Authors
